# Draft Genome Sequence of *Glycomyces fuscus* TRM 49117, Isolated from a Hypersaline Soil Sample

**DOI:** 10.1128/genomeA.01258-17

**Published:** 2017-12-14

**Authors:** Yang Wang, Xiaoxia Luo, Lili Zhang

**Affiliations:** College of Life Science, Key Laboratory of Protection and Utilization of Biological Resources in Tarim Basin of Xinjiang Production & Construction Corps, Tarim University, Alar, China

## Abstract

*Glycomyces* spp. are rare actinomycetes that are potential antibiotic producers. Here, we report the draft genome sequence of *Glycomyces fuscus* TRM 49117. This is the first genome report of a bacterium belonging to the genus *Glycomyces*. The genome information of *G. fuscus* will contribute to studies on the structure and function of antibiotics.

## GENOME ANNOUNCEMENT

In a previous study, a strain was reported to show antibacterial activity against the rare actinomycete *Glycomyces harbinensis* ATCC 43155^T^ (1). It was previously discovered that the high-temperature, drought-ridden, and high-salinity Lop Nur environment contains a large number of *Glycomyces* actinomycetes. New species may produce new metabolites and active substances. *Glycomyces fuscus* TRM 49117 was originally isolated from a soil sample from the Lop Nur salt lake in Xinjiang, China (39°35′N 89°46′E). It can tolerate a higher salt concentration than can *G. harbinensis* ATCC 43155^T^ or *Glycomyces* spp. from other environments and can grow in more than 10% ISP4 medium, and it contains a more abundant polar lipid than does *G. harbinensis* ATCC 43155^T^ or *Glycomyces* spp. from other environments (2).

The genome was sequenced using an Illumina HiSeq 2000 platform. Two different sequencing libraries, a 500-bp paired-end and 6,000-bp mate pair library, were used. Sequencing yielded 230-fold genome coverage. Assembly of all sequence reads (1,002-Mbp paired-end reads and 808-Mbp mate pair reads) applying the SOAPdenovo 1.05 software (3) resulted in a draft genome comprising 18 scaffolds, with 63 contigs with an *N*_50_ size of 193,136 bp and a longest contig of 613,903 bp. The total scaffold size was determined to be 6,556,887 bp, with a GC content of 72.84%. Genome annotation was accomplished using the Rapid Annotations using Subsystems Technology (RAST) server (4). Briefly, protein-coding genes were identified using Glimmer version 3.0 (5). Ribosomal RNAs were predicted by a sequence similarity search using BLAST against an RNA sequence database and/or using the Infernal and Rfam models (6, 7). tRNAs were predicted using tRNAscan-SE (8). The predicted coding sequences (CDSs) were translated and used to search the National Center for Biotechnology Information (NCBI) nonredundant (NR), UniProt, TIGR-Fam, Pfam, PRIAM, KEGG, COG, Swiss-Prot, TrEMBL, GO, and InterPro databases. Other noncoding genes were predicted using Infernal 1.0 (9). Additional gene prediction analysis and functional annotation were performed within the Integrated Microbial Genomes-Expert Review (IMG-ER) platform (10). Gene prediction revealed 5,666 protein-coding genes, 9 rRNA operons, and 74 tRNA genes. Among these protein-coding sequences, 5,582 (98.52%) have been assigned to a Clusters of Orthologous Groups (COG) functional category (11).

The complete genome sequence includes a number of gene regions devoted to the biosynthesis of secondary metabolites. The *G. fuscus* TRM 49117 genome sequence was scanned by antiSMASH (12), and we found 4 type I and 1 type II polyketide biosynthesis gene clusters and 6 nonribosomal peptide synthetase biosynthesis gene clusters. Also, we found 5 gene clusters for a bacteriocin-like bioactive substance, which is usually produced by Gram-positive bacteria and can inhibit other closely related Gram-positive bacteria (13).

The *G. fuscus* TRM 49117 genome information will serve as a valuable reference to elucidate the potential of hypersaline-derived *Glycomyces* strains as promising sources for bioactive secondary metabolites. The *G. fuscus* TRM 49117 genome sequence provides us the opportunity to further understand the evolutionary relationship between *Glycomyces* strains and to explain the physiological mechanism for its growth in hypersaline soil. The genome sequence of *G. fuscus* was determined, and the genes possibly involved in the antibacterial activity were determined.

### Accession number(s).

The *G. fuscus* TRM 49117 draft genome sequence was deposited in the NCBI database under the accession no. NYMS00000000. The version described in this paper is the first version.
